# A Randomized Controlled Trial on the Effect of Tapentadol and Morphine on Conditioned Pain Modulation in Healthy Volunteers

**DOI:** 10.1371/journal.pone.0128997

**Published:** 2015-06-15

**Authors:** Chris Martini, Monique van Velzen, Asbjørn Drewes, Leon Aarts, Albert Dahan, Marieke Niesters

**Affiliations:** 1 Department of Anesthesiology, Leiden University Medical Center, Leiden, The Netherlands; 2 Mech-Sense, Department of Gastroenterology & Hepatology, Aalborg University Hospital, Aalborg, Denmark; Center for Rheumatic Diseases, INDIA

## Abstract

**Background:**

Modulatory descending pathways, originating at supraspinal sites that converge at dorsal horn neurons, influence pain perception in humans. Defects in descending pain control are linked to chronic pain states and its restoration may be a valuable analgesic tool. Conditioned pain modulation (CPM) is a surrogate marker of descending inhibition that reduces the perception of pain from a primary test stimulus during application of a conditioning stimulus. Here the effects of the analgesics tapentadol, a combined mu-opioid receptor agonist and noradrenaline reuptake inhibitor, and morphine, a strong mu-opioid receptor agonist, were tested on CPM in a randomized, double-blind, placebo-controlled crossover trial in 12 healthy pain-free volunteers, to understand possible differences in mechanism of action between these opioids.

**Methods and Results:**

On three occasions CPM responses were obtained 60-90 and 120-150 min following intake of tapentadol (100 mg immediate release tablet), morphine (40 mg immediate release tablet) or placebo. At both time points, CPM was detectable after treatment with placebo and tapentadol (peak pain ratings reduced by 20-30% after application of the conditioning stimulus) but not after morphine. Compared to placebo morphine displayed significantly less CPM: mean treatment difference 18.2% (95% CI 3.4 to 32.9%) at 60-90 min after drug intake and 19.5% (95% CI 5.7 to 33.2%) at 120-150 min after drug intake (p = 0.001). No difference in CPM between placebo and tapentadol was detected: mean treatment difference 1.5% (95% CI -11.6 to 14.6%) at 60-90 min after drug intake and 1.5% (95% CI -16.0 to 18.9%) at 120-150 min after drug intake (p = 0.60).

**Conclusions:**

Our data show that in volunteers morphine affects CPM, while tapentadol was without effect despite identical experimental conditions. These data confirm that tapentadol’s main mechanism of action is distinct from that of morphine and likely related to the effect of adrenergic stimulation on descending controls.

**Trial Registration:**

Netherlands Trial Register NTR2716

## Introduction

Pain perception is dependent on several modulatory mechanisms that transform afferent nociceptive input into the sensation of pain [1]. Such endogenous pain modulatory mechanisms may be inhibitory or facilitatory and determine the intensity of the painful sensation (ranging from no pain to extremely painful) following a noxious event. An inhibitory mechanism that may be activated under experimental conditions is conditioned pain modulation or CPM; CPM is a surrogate biomarker of endogenous modulation of pain [2–5] and is the human equivalent of diffuse noxious inhibitory controls or DNIC in animals. In the case of DNIC, a spinal-medullary-spinal feedback loop (without the involvement of higher brain centers) is engaged, causing the inhibition of a primary noxious stimulus by a spatially separated secondary noxious event due to inhibition of sensory spinal dorsal horn neurons [6]. In contrast to DNIC (in animals), CPM (in humans) equires activation of supraspinal pathways. For example, in a recent study, Nahman-Averbuch et al. [7] show in an fMRI study that CPM is associated with reduced activity in nociceptive brain regions such as the thalamus, insula and secondary somatosensory cortex and brainstem. Alike DNIC, CPM is tested by applying a primary (test) nociceptive stimulus on one part of the body simultaneously with a secondary (conditioning) tonic nociceptive stimulus on a body part remote from the primary site [1–5]. Data from healthy young volunteers invariably show that the conditioning stimulus inhibits the pain perceived from the test stimulus [4, 8,9]. The current notion is that CPM engages top-down inhibitory pathways that originate at higher sites and converge at dorsal horn neurons to suppress incoming afferent nociceptive information [10, 11].

Apart from CPM, offset analgesia (OA) is another psychophysical paradigm, which enables testing of endogenous modulation of pain [7,8,12]. In contrast to CPM, OA reflects temporal filtering of nociceptive information, and causes robust analgesia upon a slight decrease (offset) in noxious stimulus intensity. OA and CPM differ significantly: (1) fMRI studies show that OA is associated with an *increase* in activity in certain brain areas such as the anterior insula, dorsolateral prefrontal cortex and brainstem [7]; (2) There is no correlation between OA and CPM responses [7,8]; (3). When affected in chronic pain states, CPM but not OA is improved by pharmacotherapy [13,14]. These findings indicate that OA and CPM engage two distinct endogenous modulatory mechanisms.

The inability to recruit CPM-related descending inhibitory pain pathways is thought to play an important role in the development of chronic pain. For example, patients with a less efficient or absent CPM have a higher probability of chronic pain following thoracic, abdominal or obstetric surgery [15–17]. Similarly, rodents with the genetic predisposition to effectively engage descending inhibition are better protected against the development of chronic pain following peripheral nerve damage compared to animals with a lesser ability to do so [18]. Furthermore, in a variety of painful conditions (including complex regional pain syndrome; diabetic-, sarcoidosis- and chemotherapy-induced polyneuropathy; fibromyalgia; chronic pancreatitis) impaired CPM responses have been observed [13,19–24].

The observation of a link between defects in descending pain control, as tested by CPM, and development and chronification of pain suggests that restoration of descending pain control may be a valuable analgesic tool. Several analgesics have the potential to activate or reinforce descending inhibition as they interact favorably with the neurotransmitter systems involved in descending pain pathways. Most important neurotransmitters include endogenous opioid peptides, noradrenaline (NA) and serotonin and it is thought that their release can produce pain inhibition [10,11]. Tapentadol is a relatively new analgesic agent that is believed to activate descending inhibitory pain pathways [25]. Tapentadol is a combined μ-opioid receptor (MOR) agonist and inhibitor of the neuronal reuptake of the α_2_-adrenergic receptor agonist NA [26]. Compared to morphine it displays weak agonist activity at the μ-opioid receptor (MOR), however, due to synergy between the two mechanisms of action, tapentadol has strong antinociceptive and antihypersensitive effects [27], possibly via enhancement of descending inhibitory pathways.

We performed a randomized, double blind, placebo-controlled crossover study in healthy (pain free) volunteers to quantify the effect of tapentadol and morphine on CPM. Morphine, in contrast to tapentadol, is a full MOR agonist without significant effect on NA reuptake. Previous data suggest that morphine reduces CPM responses in volunteers [28]. We hypothesize that taken the differences in mechanisms of action of morphine and tapentadol and the earlier observation with morphine, these two analgesics behave differently in their effect on CPM with a reduction in CPM by morphine and an increase in CPM by tapentadol.

## Materials and Methods

### Ethics statement and trial registration

Adult volunteers were recruited to participate in this double blind, randomized, placebo-controlled, crossover study that was performed at Leiden University Medical Center. All participants gave written informed consent to participate in the trial. Approval of the protocol and consent procedure was obtained from the LUMC Medical Ethics Committee and the Central Committee on Research involving Human Subjects (CCMO, The Hague, The Netherlands). Trial registration was at www.trialregister.nl under number NTR2716 (Feb 2011). The protocol and CONSORT checklist are available for review (see S1 Protocol and S1 CONSORT Checklist).

### Participants

Twelve healthy Dutch-speaking (non-smoking) volunteers of either sex, aged ≥18 years with a body mass index < 30 kg/m^2^, were enrolled in the study Exclusion criteria included: known drug allergies, current use of legally prescribed medication; present or past illicit substance use; present or past alcohol abuse; history of mental illness; pregnancy and/or lactation; any other condition that in the opinion of the investigators would compromise the well-being of the subject when participating in this trial. All volunteers were instructed to abstain from solids and liquids during the 8 hours before dosing.

### Study design

After arrival in the laboratory and an initial practice session, the temperatures of the test and conditioning stimulus were determined. Next the study medication was ingested. CPM responses were obtained 60–90 and 120–150 min after drug intake.

#### Treatment

The subjects received placebo (cellulose; fabricated by the local pharmacy), morphine immediate release 40 mg (Sandoz BV, Almere, The Netherlands), and tapentadol immediate release 100 mg (Grünenthal BV, Breukelen, The Netherlands) tablets on separate occasions. The order of administration was random. At least two weeks was allowed between treatments for washout. The dose of tapentadol IR was based on the observation that in postsurgical dental pain an oral dose of 100 mg IR caused pain relief for 4 hours (peak effect after 1–1.5 h) [29]. The dose of morphine is based upon a 2.5-fold greater potency of morphine compared to tapentadol (data provided by the manufacturer and [30]).

#### Test stimulus

The test stimulus was a noxious heat stimulus, administered via a calibrated 3×3 cm thermal probe connected to the Pathway Neurosensory Analyzer (Medoc Ltd., Ramat Yishai, Israel). The thermode was placed on the volar side of the lower part of the (non-dominant) upper extremity. During noxious testing the subjects scored their pain intensity rating on an electronic slider, which ranged from 0 (no pain) to 100 (worst pain imaginable). These data on the electronic visual analogue scale (eVAS) were collected at 1 Hz for further analysis. To prevent sensitization of the skin, the thermal probe was repositioned between different zones on the forearm and ample time was allowed between testing.

The temperature of the test stimulus was determined by applying a series of heat stimuli. First the temperature was increased from 32°C (baseline temperature) by 1.5°C/s to a target temperature of 42°C and kept constant for 10 seconds. If the eVAS was less than 50 mm a next test was performed increasing the target temperature in steps of 1°C. The cut-off temperature for these series was 49°C. The lowest temperature evoking an eVAS of at least 50 mm was used during the remainder of the study. This procedure was performed, once on each experiment day, before drug administration. Consequently, small deviations in test stimulus intensity (but not test target temperature) due to the presence of the analgesic were allowed.

#### Conditioning stimulus (CS)

The CS was applied to the foot and lower leg of the subject (ipsilateral to the site of the test stimulus) and consisted of a thermal (cold water) stimulus. To generate the stimulus the subject’s lower extremity was immersed into an 84 L water reservoir. The water was produced by a rapid counter-current water-cooling system (IcyDip, IcySolutions BV, Delft, The Netherlands). Water temperatures in the range of 6°C to 18°C (in steps of 0.5°C) were offered to the subject and the highest temperature that produced an eVAS of at least 30 mm was used in the remainder of the study. After the exposure to cold water, the subject’s extremity was warmed to normal temperature using warm water collected from the counter-current outlet of the IcyDip system. There was at least 1 hour between obtaining the optimal test and conditioning stimuli.

#### Conditioned pain modulation

**Fig 1:**
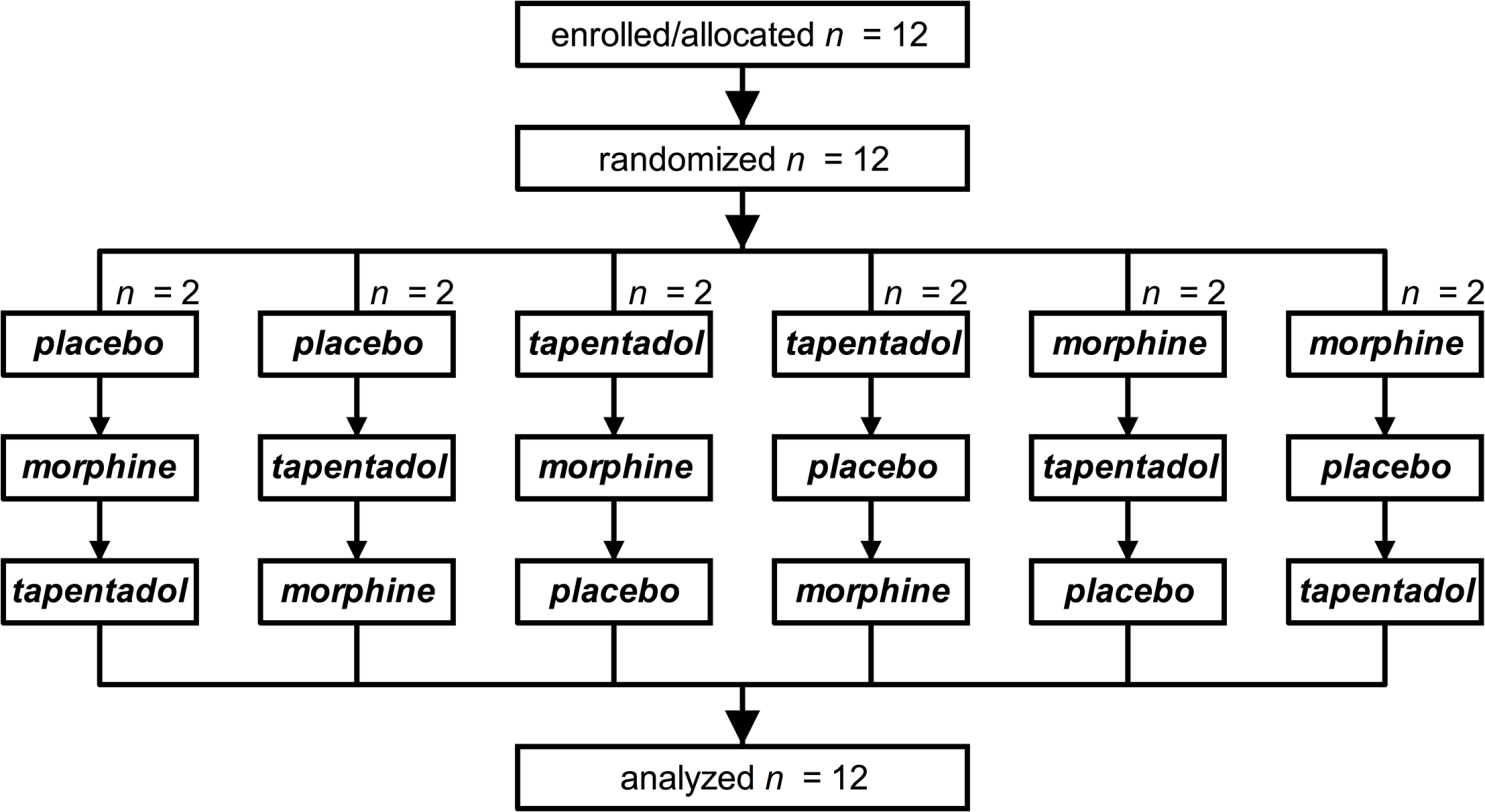
The setup used to induce Conditioned Pain Modulation (CPM). The volunteer sits on the bed while a thermal test stimulus is applied to the arm *via* a 3×3 cm thermode (insert top right). The leg is placed in a bucket filled with cold water (*ie*. the conditioning stimulus) and the subject scores the heat pain to the arm using an electronic visual analogue scale (insert top middle). The cold water is applied via the Icydip system; the heat pain via Medoc’s pathway system.

We applied the method of King *et al*. [19,31] to measure CPM, which was used successfully in previous studies from our laboratory [8,14,22]. In short, the thermal probe was applied to the volar side of the (non-dominant) underarm and its temperature was increased from baseline 32°C (temperature close to skin temperature) to the test temperature (at 1.5°C/s) and was kept constant for 30 s. Next, the temperature was rapidly lowered (at 6°C/s) to baseline. Each heat test was applied 3 times (with a 3-min rest between tests) after which this same sequence was repeated but now combined with the conditioning stimulus (immersion of foot and leg in cold water). The conditioning stimulus was applied 25 s before the test stimulus and continued until the end of the test stimulus. During all tests the subjects were instructed to rate the pain intensity of the test stimulus (and not the conditioning stimulus) using the eVAS slider. The whole procedure lasted no longer than 30 min. See Fig 1 for an overview of the setup used to induce CPM.

### Randomization and blinding

Non-restricted randomization and allocation (1:1:1) was performed by the local pharmacy using a computer-generated randomization list. To ensure blinding of both investigators and participants, all tablets were recapsulated into identical capsules in form and taste. The capsules were dispensed to the research team on the morning of the study in containers marked by subject number and visit number. The investigators remained blinded to treatment until all data were analyzed.

### Data analysis

The study was powered to detect a difference in CPM of 20 ± 15% (mean ± SD, with α = 0.05 and 1-β > 0.95) between active treatment (morphine or tapentadol) and placebo (SigmaPlot v12.0, Systat Software Inc., San Jose, CA) [8,22]. To quantify the magnitude of CPM, the average peak eVAS without (from 3 tests) and with CS (from 3 tests) were calculated. Next, CPM responses were calculated to correct for variations in peak response between sessions and subjects using the formula [8,14,22]:

[Peak eVAS(without CS)–Peak eVAS(with CS)]/[Peak eVAS(without CS)]×100% (1)

Temperatures of test and conditioning stimuli of the three treatment groups were compared by one-way analysis of variance. The effect of the conditioning stimulus on peak eVAS was tested by two-tailed *t*-test. To test the effect of treatment on CPM responses, a mixed effect model was applied to the data (factors: treatment, time and time×treatment) using package “ImerTest” of the R software package (version 2.8, R-project; http://www.r-project.org); *post hoc* a linear mixed model was fit to the data to assess the significance of difference between active treatment and placebo. p-values < 0.05 considered significant. Data are presented as mean ± SEM unless otherwise stated.

## Results

**Fig 2:**
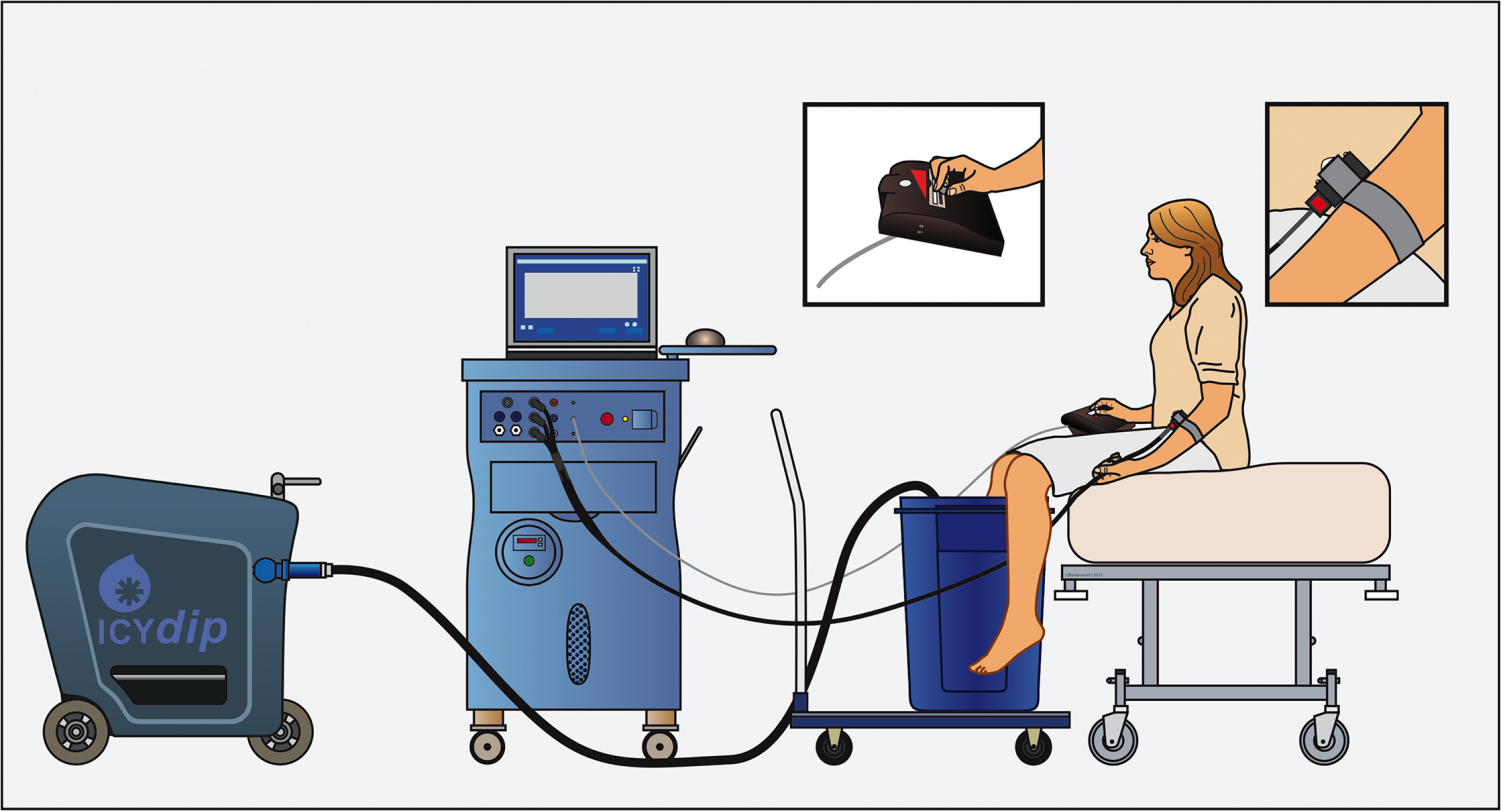
Consort flow diagram. The test stimulus temperatures (heat pain) as determined prior to treatment averaged to 47 ± 0.5°C, 46.6 ± 0.5°C and 46.9 ± 0.5°C on placebo, morphine and tapentadol experimental sessions (one-way anova: NS). The target conditioning stimulus temperatures were 7.9 ± 0.5°C, 8.1 ± 0.6°C and 8.3 ± 0.6°C on placebo, morphine and tapentadol experimental sessions (one-way anova: NS).

Seven men and five women were enrolled from March 2011 to June 2011, aged 26 ± 1 years and with a body mass index of 23 ± 1 kg/m^2^ (mean ± SD). All twelve volunteers were randomized and dosed and their data were fully analyzed according to protocol (Fig 2). None of the volunteers experienced any side effects following treatment with placebo, morphine and tapentadol, except for one female subject who became nauseous after tapentadol and morphine treatment and vomited after morphine.

### Effect of the conditioning stimulus of the eVAS of the test stimulus

**Fig 3:**
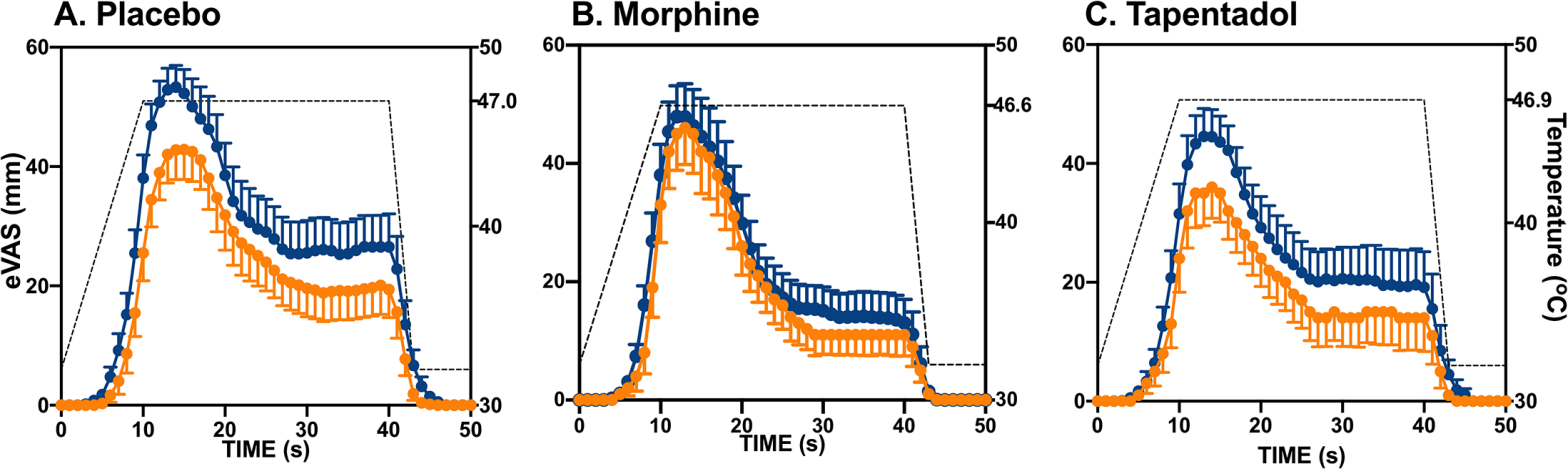
Mean eVAS responses following treatment with placebo (A), oral morphine immediate release 40 mg (B) and oral tapentadol immediate release 100 mg (C) at 60–90 min after drug intake. The blue dots are the responses to the test stimulus without conditioning stimulus, the orange dots the responses to the test stimulus with conditioning stimulus. The broken line is the mean temperature of the test stimulus. Values are ± SEM.

**Fig 4:**
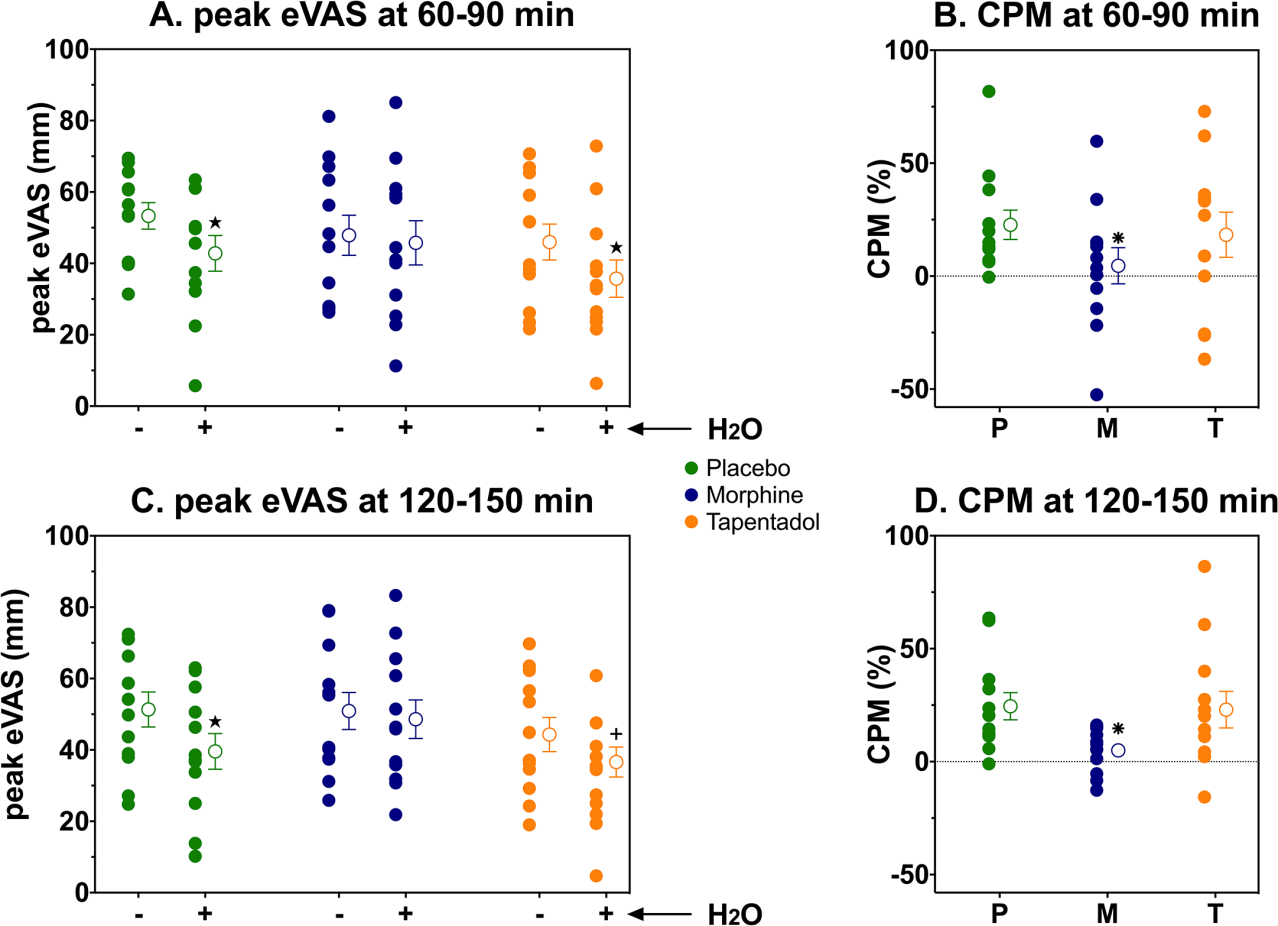
eVAS and CPM responses at 60–90 min (A and B) and 120–150 min (C and D) after drug intake. **A and C.** Mean peak eVAS responses without (–) and with (+) conditioning stimulus for placebo (green), morphine (blue) and tapentadol (orange). **B and D.** CPM responses observed following placebo, morphine and tapentadol treatment. Individual data and the mean ± SEM are presented. ★ p < 0.001, * p = 0.001, + p = 0.03.

In Fig 3 the mean eVAS responses to the test stimulus before (blue dots) and after (orange dots) application of the conditioning stimulus are given obtained at 60–90 min after drug intake. At that time period, a significant decrease in peak eVAS for placebo (peak eVAS without CS = 53 ± 4 mm *vs*. peak eVAS with CS = 43 ± 5 mm, p < 0.001) and tapentadol (45 ± 5 mm *vs*. 36 ± 5 mm with CS, p < 0.001) was observed, but not for morphine (48 ± 5 mm *vs*. 46 ± 6 mm, p = 0.48). Similarly, at 120–150 min after drug intake, a significant decrease in peak eVAS for placebo (peak eVAS without CS = 51 ± 5 mm *vs*. peak eVAS with CS = 40 ± 5 mm, p < 0.001) and tapentadol (46 ± 5 mm *vs*. 33 ± 4 mm with CS, p = 0.03) was observed, but not for morphine (51 ± 5 mm *vs*. 46 ± 6 mm, p = 0.12). See also Fig 4A and 4C.

### CPM responses

CPM responses were 22.7 ± 6.5% (placebo), 4.5 ± 8.0% (morphine) and 21.2 ± 8.9% (tapentadol) after 60–90 min and 24.4 ±6.0% (placebo), 5.0 ± 2.8% (morphine) and 23.0 ± 8.1% (tapentadol) after 120–150 min. A significant treatment effect was observed (F2,55 = 6.2, p = 0.004), but there was no time effect (F1,55 = 0.24, p = 0.62) or time×treatment effect (F2,55 = 0.072, p = 0.93). Treatment effects relative for placebo were -18.8% (95% confidence interval -29.8 to -7.8%, p = 0.001) for morphine and -2.9% (95% confidence interval -13.9 to 8.1%, p = 0.60) for tapentadol. See Fig 4B and 4D.

## Discussion

The effect of two opioid analgesics, morphine and tapentadol, on conditioned pain modulation was tested in a single population of healthy young volunteers under identical experimental and placebo-controlled conditions. The main findings of our study are: (1) In agreement with our hypothesis, a single oral dose of morphine reduces CPM responses relative to placebo by 80%; (2) In contrast to our hypothesis, a single dose of tapentadol has no effect on CPM responses compared to placebo treatment.

Tapentadol is a combined μ-opioid receptor (MOR) agonist and inhibitor of the neuronal reuptake of the α_2_-adrenergic receptor agonist NA [26]. Noradrenaline, together with other monoamines, plays an important role in mediation of analgesia *via* activation of the α_2_-adrenergic receptor at supraspinal and pre- and postsynaptic spinal sites [10,11]. Animal data indicate that the combined effect of MOR agonism and NA reuptake inhibition (the so called MOR-NRI concept) of tapentadol produces synergistic analgesia [27]. In contrast to tapentadol, morphine has no effect on NA reuptake. *In vivo* animal data of microdialysis in the ventral hippocampus show that tapentadol but not morphine produces a dose-dependent increase in extracellular levels of NA [26]. In mice lacking the MOR, tapentadol’s analgesic effect is reduced by about 50% in a model of acute thermal pain and by about 20% in a model of neuropathic pain [32]. In contrast, morphine is without any antinociceptive effect in this MOR knockout mouse strain.

In the current study, we tested a single dose of tapentadol (100 mg) and a single dose of morphine (40 mg). The doses chosen were based on equianalgesia data from animal and human studies. For example, in mice the ED_50_ in the hot plate assay is 11.8 and 4.7 mg/kg for tapentadol and morphine, respectively [26]; and in the treatment of patients with cancer pain the equianalgesic tapentadol/morphine dose ratio is 2.5 [30]. Although our data indicate analgesia from morphine and tapentadol (see Figs 3 and 4), our study was not designed to assess (equi)analgesia between treatments and the phasic heat pain may not be the best experimental model to evaluate potency of opioids [33].

The reduction of CPM with morphine is in agreement with an earlier observation of Le Bars et al. from 1992 in healthy volunteers [28]. To the best of our knowledge our study is the first to re-assess the effect of morphine on CPM in volunteers. After 0.05 mg/kg morphine, Le Bars et al. observed a complete loss of depression of the spinal nociceptive R_III_-reflex (elicited by electrical stimulation of the sural nerve) by a contralateral conditioning temperature stimulus. The inhibition returned upon administration of the opioid antagonist naloxone. Animal data, showing similar effects on DNIC, suggest that the effect of morphine on DNIC originates at supraspinal sites [34]. The data from Le Bars et al. and ours provide robust proof for an inhibitory effect of morphine on CPM in volunteers without pain.

It is of interest to speculate on the mechanism through which morphine reduces CPM. A specific role form activation of the μ-opioid receptor would be expected in this respect. However, other opioids that have an effect on the μ-opioid receptor (such as oxycodone [9]) do not affect the CPM response as tested within 1–3 hours after the administration of the opioid. Furthermore, three of six human studies on the effect of naloxone or naltrexone on CPM found no effect of opioid receptor blockade on CPM [35–37], while three others showed a reduction of CPM [31, 38, 39]. This suggests a limited role for the opioid-receptor system in the reduction of CPM. Alternatively, the loss of CPM by morphine may be related to a morphine-specific property such as due to an effect of the pronociceptive metabolite morphine-3-glucuronide. This metabolite appears rapidly in plasma upon the administration of morphine [40]. Overall, the data on morphine are confusing and presently offer no definite conclusion regarding the involvement of opioid receptors in the development of descending (inhibitory or facilitatory) controls in humans.

We did not observe an increase in CPM by tapentadol. Since monoaminergic receptors play an important role in descending pain inhibition [10,11], we expected an increase in CPM. Multiple, nonexclusive mechanisms may have played a role in our finding: (i) In the population of pain-free volunteers, the single administration of tapentadol was insufficient to have a clinical significant effect on neuronal NA reuptake and/or the dose was too low. However, after this single dose we did observe an analgesic effect on peak eVAS (Figs 2 and 3), which we relate to the synergistic effect of MOR activation and NA reuptake inhibition; (ii) Possibly the CPM responses of our healthy volunteers were at their (near) physiological maximum, and a further increase is difficult to attain. In contrast to healthy volunteers, in a recent protocol we observed that in neuropathic pain patients with absent CPM responses, a single treatment with 100 mg immediate release tapentadol treatment did activate CPM to values of similar magnitude as observed in the current study after placebo and tapentadol treatment, while intravenous morphine was without effect in this similar patient population [22]; (iii) We cannot exclude that morphine’s negative impact on CPM is an opioid receptor-related effect. If so, the apparent absence of effect of tapentadol on CPM may be related to two opposing effects on CPM, one negative (related to an opioidergic effect) and one positive (due to spinal adrenergic stimulation) with a net zero effect. This seems an attractive explanation of our findings and is in agreement with animal data [26,27,32,34]. Still, further studies are needed to understand the mechanism of tapentadol’s absence of effect on CPM in healthy volunteers. For example, specific α_2_-adrenergic and opioid receptor blockers (*eg*. yohimbine and naloxone) may be used to separate MOR and adrenergic effect on CPM during tapentadol treatment.

Just few earlier studies addressed the effect of opioids on CPM responses in human volunteers [8,28,41]. Comparison with our study is difficult due to differences in dosage, administration route, and dissimilarities in methods to induce CPM. Hence, a significant part of the variability observed in study outcomes may be related to methodological issues. An interesting example is the study by Arendt-Nielsen et al, who studied the effect of fentanyl (25 μg/h) and buprenorphine (20 μg/h), administered by a transdermal patch formulation, on CPM responses after 24, 48 h and 72 h of treatment [41]. They observed an increase in CPM at all time points. Differences with our study include the route of administration (transdermal *vs*. oral), dose, duration of administration (continuous *vs*. single delivery), and CPM protocol. Arendt-Nielsen et al. used pressure pain tolerance threshold (test stimulus) applied to the left arm before, during and after immersion of the left hand into a fixed 3°C water bath (conditioning stimulus). Setting the difference in study CPM protocol aside, these data suggest that sustained but not single deliveries enhance CPM responses in human volunteers. Possibly, the secondary effect of these opioids on descending control via activation of off-cells in the medulla and release of noradrenaline in the spinal cord takes time to develop [10,11,22], and days rather than hours of treatment are required before the CPM increases above baseline levels. Whether this is also true for morphine requires further study. In order to overcome the issue of differences in CPM protocol, which is a general observation that relates to all studies on CPM, adoption of a standardized CPM protocol is required [42].

One caveat of our study is that we did not collect plasma levels of morphine and tapentadol. We refrained from blood sampling as this could have affected the study outcome and blood sampling *per se* is not a good marker of drug effect taken the sometimes relatively long and variable blood-effect-site equilibration half-life (*ie*. passage of the drug to central sites) of opioids such as morphine and tapentadol [40]. By performing CPM tests at multiple times in the first three hours following drug intake, we contend that we captured the peak drug effect on CPM for both morphine and tapentadol.

## Conclusions

Our data show that, in agreement with earlier findings, a single oral administration of morphine has a negative impact on the endogenous modulation of nociceptive stimuli in healthy young adult volunteers. In contrast, a single oral administration of tapentadol was without effect on CPM despite the fact that studies were performed under similar controlled conditions in the same study population. These data confirm that tapentadol’s main mechanism of action is distinct from that of morphine and likely related to the tapentadol-induced effect of adrenergic stimulation on descending controls.

## Supporting Information

S1 CONSORT Checklist (DOC)

S1 Protocol This is the Study protocol. (PDF)

S1 File This file contains the raw data set. (XLSX)
