# Supplementary material for: A Randomized Controlled Trial on the Effect of Tapentadol and Morphine on Conditioned Pain Modulation in Healthy Volunteers
Source: PLoS One. 2015 Jun 15;10(6):e0128997. doi: 10.1371/journal.pone.0128997 (PMC4467981; doi:10.1371/journal.pone.0128997)

Protocol P_TPT_2010
Date: September 29 – 2010, version 1.0

Influence of Tapentadol on endogenous modulation of pain in chronic neuropathic pain patients and healthy volunteers
–the TPT study–

Investigators:
Marieke Niesters, Medical and Biomedical Sciences student
Martin Bauer MD, anesthesiologist
Leon Aarts MD PhD, anesthesiologist
Albert Dahan MD PhD, anesthesiologist (PI)
Department of Anesthesiology, Leiden University Medical Center

Elske Hoitsma MD PhD, neurologist
Diaconessenhuis, Leiden

External Subjects Advisor:
Elise Sarton, MD, PhD, anesthesiologist
Department of Anesthesiology, LUMC

Address for correspondence:
Prof. dr. A. Dahan
Dept. of Anesthesiology
LUMC, P5-Q, 2300 RC Leiden, The Netherlands
FAX: +31 71 526 6230 e-mail: a.dahan@lumc.nl

Contents

1. Introduction and study aims
2. Patients and volunteers
Patient inclusion criteria.
Volunteer inclusion criteria.
Patient and volunteer exclusion criteria.
Recruitment.
Informed consent.
Medical examination.
Pre-study requirements.
Reimbursement.
Withdrawal of an individual subject.
3. Study Design
Pain measurement.
DNIC and Offset Analgesia.
Treatment.
4. Treatment
Morphine
Tapentadol
Placebo
5. Side effects
Respiratory depression.
Nausea.
Emergency.
6. Sample size and statistics
Sample size.
Within group comparison.
Between group comparison.
7. Educational grant
8. References
9. Information for volunteers
10. Informed consent
11. Advertisement
12. Addendum: Declaration by the Investigator + Signature

1. Introduction and study aims

Endogenous modulation of pain. Pain perception is modulated *via* facilitatory and inhibitory control systems. Inhibitory control is most important to chronic pain patients as there are strong indications that failed inhibition constitutes a predisposition to acquired chronic pain. Various systems involved in inhibitory control have been demonstrated over the years. Two systems seem important: (1) top-down inhibition of afferent noxious information by endogenous analgesia originating in the periaquaductal grey (PAG) and affecting pain perception *via* descending pathways; (2) bottom-up activation of pain modulatory systems *via* activation of spino-bulbo-spinal loops originating in the dorsal horn of the spinal cord. The effect that the latter system has on pain perception is called Diffuse Noxious Inhibitory Control (**DNIC**). The two systems are interconnected and DNIC is considered a bottom-up activation of the pain modulatory mechanism, as part of the descending endogenous analgesia system. DNIC dysfunctions or is less efficacious in various complex chronic pain states, such as irritable bowel syndrome, chronic headache, fibromyalgia and temporomandibular disorder.

Offset analgesia (**OA**) is another expression of the endogenous analgesia system and is evoked by noxious stimulation, in order to reduce (or control) the perception of the noxious event. Offset analgesia becomes apart when an even more painful stimulus occurs briefly during prolonged painful stimulation. Due to activation of the endogenous opioid system the prolonged stimulation is perceived less painful after the intense noxious stimulus than therefore. In a current study (P09.107) we observed that patients with neuropathic pain have a delayed OA or sometimes even absent OA, suggesting a crucial role of pain pathways involved in OA in the development of chronic pain.

Neuropathic pain. Neuropathic pain is a form of chronic pain due to an evident nerve lesion from trauma (incl. surgical trauma), diabetes (small fiber neuropathy), infection (incl. HIV), chemotherapy, etc. The primary sensation is a burning pain coinciding with areas of hyperalgesia and allodynia. In the current study we will focus on patients with chronic neuropathic pain caused by diabetes.

Tapentadol and endogenous modulation of pain. Tapentadol is a centrally acting analgesic with two mechanisms of action: a μ-opioid receptor agonism and noradrenaline (NA) reuptake inhibition. Although the binding of tapentadol to the μ-opioid receptor is weaker than that of morphine its analgesic action is similar to that of morphine due to the (synergistic) effect of the second mechanism (i.e., NA reuptake inhibition). NA plays a role in the endogenous descending pain inhibitory system. Especially at descending pathways NA reuptake inhibition plays a crucial role at the spinal level to reduce chronic

neuropathic pain. Hence it is to be expected that tapentadol has a modulatory role on DNIC and OA and consequently will ameliorate pain in chronic neuropathic pain patients. This is the main focus of our current study.

The aims of our study are:

1. Measure DNIC and offset analgesia in neuropathic pain patients;

2. Compare DNIC and offset analgesia in chronic pain patients with DNIC and offset analgesia in healthy volunteers;

3. Assess the effect of oral tapentadol on DNIC and offset analgesia relative to placebo and morphine.

2. Patients and volunteers

We will study healthy volunteers (*n* = 12) and patients with small-fibre neuropathy (*n* = 12). The patient population will consist of patients referred to the LUMC pain treatment center or neurology outpatient clinic (LUMC and Diaconessenhuis). We will study healthy volunteers of either sex matched for age and sex relative to the neuropathic pain patient populations.

Patient inclusion criteria. (i) Patients diagnosed with small-fiber neuropathy or according to the guidelines of the IASP or other professional pain societies (eg., Netherlands Society of Anesthesiologists); (ii) a pain score of 5 or higher; (iii) age between 18 and 75 years; (iv) being able to give written informed consent.
Volunteer inclusion criteria. Healthy volunteers in the age range 18-75 years of either sex.

Patient and volunteer exclusion criteria. (i) Unable to give written informed consent; (ii) medical disease such as pulmonary, renal, liver, cardiac, gastro-intestinal, vascular (incl. hypertension) disease; (iii) allergy to study medication; (iv) use of strong opioids; (v) use of benzodiazepines; (vi) history of illicit drug abuse or alcohol abuse; (vii) history of psychosis; (viii) epilepsy; (ix) raised intracranial pressure;(x) pregnancy and/or lactation.

Recruitment. The subjects will be recruited after approval of the protocol by the Medical Ethics Committee of the Leiden University Medical Center (Commissie Medische Ethiek). Volunteers will be recruited via an advertisement in one of the university magazines, local newspaper or a flyer placed in the hospital library, restaurant and student facilities. Patients will be recruited via the pain treatment center or neurology outpatient clinic.

Informed consent. Informed verbal and written consent will be obtained from each subject.

Medical examination. Prior to the study, all volunteers will be subjected to a physical examination and a medical history. This will be done at the preoperative anesthesia clinic (POS, unit 2, second floor, K wing). Patient examination is performed at the pain treatment center or neurology outpatient clinic.

Pre-study requirements. All subjects will be asked to refrain from food and drinks for at least 8 hours prior to the study. Furthermore no grapefruit juice is allowed for 6 days prior to the study; and no alcohol/ coffee/ tea/ chocolate for the 24-h prior to the study.

Reimbursement. All participants will receive reimbursement of euro 80. Subjects may choose to end the study at any time. They will then be paid euro 10 per hour spent in the laboratory.

Withdrawal of an individual subject. Subjects can decide to leave the study at any time, for any reason if they wish to do so, without any consequences. The responsible investigator can also decide to exclude a subject if by continuing participation the subject's well being is harmed in any way. Subjects can also be excluded in case of protocol violations and non-compliance. In case of a severe adverse event, a medical examination will be performed, and hematological and blood chemistry laboratory tests or other specific examinations will be performed.

In case of dropping out from the study at the subject's own request, the subject is asked permission for using the data already collected. The subject is allowed to decline this request, without giving any reason, and again without any consequences. When permission is not granted to use already available data, this specific data is deleted from the database and any paperwork will be disposed of.

3. Study Design

Pain measurement (see also figure 1 and 2). The Visual Analogue Scale (VAS) will be used to quantify pain intensity in response to a noxious thermal stimulus. The thermal stimulus will be applied on the volar side of the forearm using the thermal probe (a 3×3 cm thermode) of the TSA-II NeuroSensory Analyzer (Medoc Ltd, Ramat Yishai, Israel). This is a computer-controlled device capable of generating highly reproducible thermal stimuli. VAS will be measured electronically (eVAS) using a slide potentiometer (length = 10 cm) that can be moved from the left (0 or no pain) to the right (10 or most intense pain imaginable). Using a hand the subject can move the slide during the heat stimulator test. The eVAS is recorded and collected on disk for further analyses.

The heat stimuli that will be applied are given in figure 1. Each subject will undergo a series of stimuli to measure DNIC and a series to study offset analgesia. The stimuli are intended to evoke an eVAS of 5-6 cm. To assess the temperature needed to reach that value various short (6 s) test stimuli will be applied in the range from 42 to 50 ^{o}C. The study will be performed with the lowest stimulus strength (in ^{o}C) which causes an eVAS of 5-6 cm. This procedure further yields a reliable estimate of the subject's pain threshold.

Finally, in order to overcome any adaptation or sensitization we will vary the location of the thermode among 3 locations: stimulus 1 $\rightarrow$ lower arm location 1, stimulus 2 $\rightarrow$ lower arm location 2, stimulus 3 $\rightarrow$ lower arm location 3.

Similar tests are commonly used to study the effects of analgesics (see also www.medoc-web.com > Resource Center > Search Reference Bibliography > pharmaceutical trials). The literature indicates that a cutoff of 53°C is acceptable (see Angst et al., Anesthesiology 2004; 101: 744-752 and Fillingim et al. Anesthesiology 2004; 100; 1263-1270).

Since we will apply an immersion of one of the lower extremities in cold water, we will test the effect of three water temperatures (16, 12 and 8 ^{o}C) for 30-s in order to reach an eVAS of 2-3 cm The immersion of the feet will be alternated and the foot will be warmed with a blanket in between immersions. The highest water temperature which causes an eVAS of 2-3 cm will be used in the remained of the study.

DNIC and Offset Analgesia (figs. 1 and 2). After the assessment of the stimulus at which the experiment will be performed the subject will be randomized to continue with either DNIC or Offset Analgesia.

In the DNIC experiments, the eVAS response to heat pain stimulation of the forearm will be assessed in threefold with 3-min intervals. For each of the three tests a different part of the skin is used. Next the same series was repeated but now with simultaneous immersion of the foot in cold water. Twenty-five s after foot immersion the heat stimulus will be applied. Just prior to the heat stimulus we will ask the subjects to rate the pain from immersion in cold water (VAS cold pain intensity).

After a 30-min rest period, three consecutive tests will be performed studying offset analgesia, again, with 3-min intervals and with each test on a different part of the skin.

The study sequence is given in the table. After baseline values have been obtained the tablet with the test drug will be given by oral route (with 100 ml of water): Tapentadol 100 mg IR per os; Morphine 35 mg IR per os, placebo). Nest the first set of OA and DNIC responses will be obtained. Subsequently the subjects will rest for 90 min and the second (test) set of responses are obtained. In patients we will ask for the VAS of their spontaneous pain. During and at the end of the study we will ask the patients to rate their spontaneous pain.

Table: sequence of events during one test day

BASELINE ASESSMENT → TREATMENT → **OA – DNIC** (in random order) →
(20-30 min) (duration 1 h)

→ 90 min pause → **OA – DNIC** (in random order)
(duration 1 h)

Treatment
Tapentadol 100 mg IR po
OR
Morphine 40 mg IR po
OR
Placebo tablet PO

All subjects (patients and volunteers) will be tested three times, once on tapentadol, once on morphine and once on placebo. Between sessions there will be at least 1 week. The treatment sequence will be randomized and blinded to the subject (i.e., single blind design).

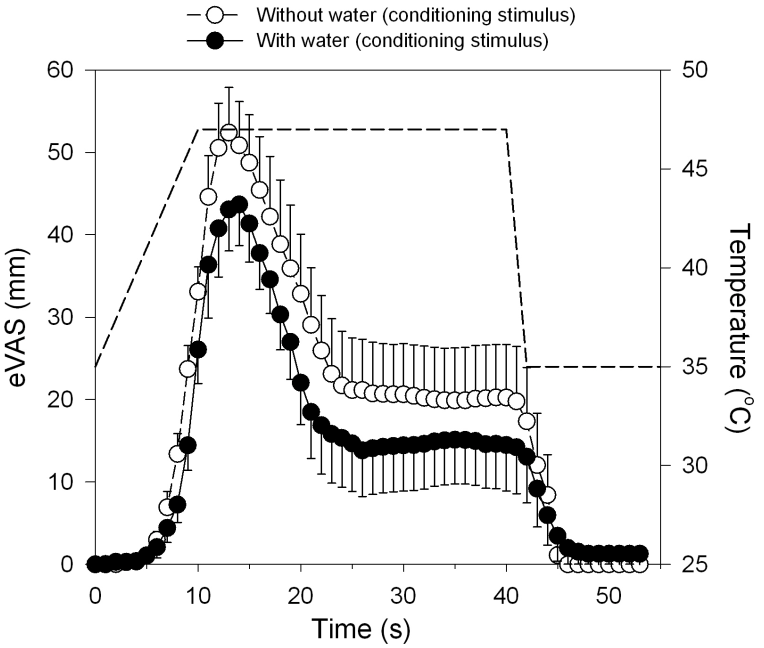

Figure 1. DNIC as tested in 10 healthy volunteers (P09.107). Open symbols denote the eVAS effect of a heat pain stimulus from 35 to 47 °C for 30 s. The closed symbols denote the response to the same heat stimulus but now with a conditioning stimulus (ie foot and lower leg in a cold water bath). The eVAS response decreased significantly, a measure of DNIC.

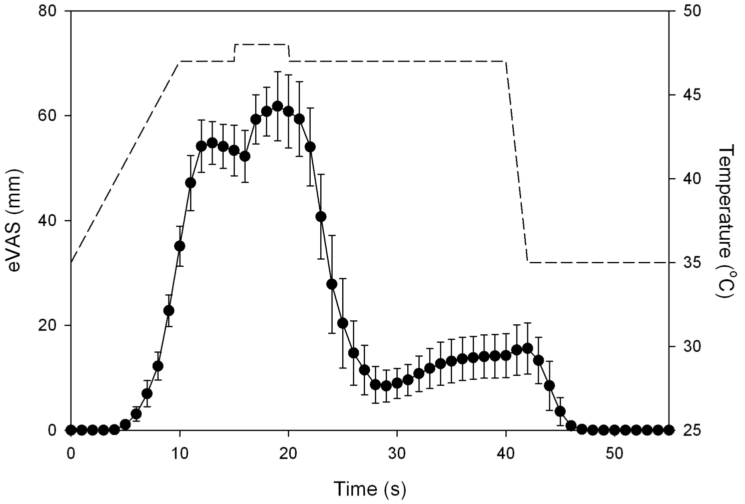

Figure 2. Offset analgesia (OA) as tested in 10 healthy volunteers (P09.107). The symbols denote the eVAS effect of a heat pain stimulus from 35 to 47 °C for 5 s, followed by a 1 °C increase for another 5 s and a subsequent decrease by 1 °C for a final 20 s. The eVAS response following the 1 °C decrease in temperature is much larger than the increase in eVAS following the 1 °C increase, a measure of OA.

4. Treatment

Morphine. Morphine is a μ-opioid receptor agonist used for the treatment of acute and chronic pain. It is the most frequently used opioid in the treatment of perioperative acute pain in the dosing range from 0.1 – 0.2 mg/kg. For chronic pain tye ose ranges from 20-120 mg per os. Side effects include nausea/vomiting, respiratory depression (see below), euphoria, dizziness, bradycardia, histamine release. We have ample experience with the use of morphine in experimental studies.

DOSE = 40 mg oral as a tablet (IR formulation).

Tapentadol. Tapentadol is a centrally acting analgesic with two mechanisms of action: a μ-opioid receptor agonism and noradrenaline (NA) reuptake inhibition. Although the binding of tapentadol to the μ-opioid receptor is weaker than that of morphine its analgesic action is similar to that of morphine due to the (synergistic) effect of the second mechanism (i.e., NA reuptake inhibition). Tapentadol (Immediate Release tablet) is currently in the final phase of registration in the Netherlands but is registered in the US. Reported side effects include dizziness, sleepiness, dry mouth, nausea and vertigo. Since we will administer tapentadol only once we expect that side effects will be mild in our study population. All subjects will be advised to go home after the study under supervision and refrain from alcoholic beverages during the evening/night of the experimnent.

DOSE = 100 mg IR oral as a tablet.

Placebo. Placebo will be a cellulose tablet.

5. Side effects

Both study drugs are opioids and as such cause opioid related side effects. The two most important side effects are discussed (respiratory depression and sedation).

Respiratory depression. Opioid agonists have a chance of producing respiratory depression. During the study the subjects are monitored with an ECG and pulse oximeter and consequently any effect of the opioids (morphine and tapentadol) on the cardiorespiratory system will be apparent. In case of overt respiratory depression (ie apnea > 20 s) naloxone iv will be administered (repeated bolus doses of 0.04 mg, max. 0.4 mg). In case of a bradycardia 0.5 mg atropine iv will be administered. Administration of naloxone (but not atropine) will end the study.

Nausea. In case of nausea ondansentron 4 mg iv may be administered. This will not end the study.

Emergency. In case of an unexpected emergency the volunteer will be placed in the Post Anesthesia Care Unit (PACU) or recovery room and appropriate treatment will be started. The PACU is an intensive care unit within the area of the operation room center and is employed under the supervision of the Department of Anesthesiology. One bed in the recovery is always available for experimental study emergencies.

6. Sample size and statistics

Sample size. The sample size calculation is based on a within group analyses, i.e., the tapentadol effect on DNIC or OA. Assuming a tapentadol effect relative to control of 200 on eVAS AUC and SD of 250, alpha of 0.05 and a beta > 0.8, we will need 12 subjects per cohort. The power analysis was performed using the statistical package SigmaPlot (http://www.sigmaplot.com/products/sigmaplot/sigmaplot-details.php), made by Systat Software Inc (US).

Statistical Analysis. The DNIC and offset analgesia data collected prior to treatment will be discarded (they are considered test data). The eVAS data will be averaged over 1-s periods. To quantify the DNIC data, the area-under-the-curve (AUC) of each eVAS response curve is calculated. A linear mixed model is used to compare the AUCs without and with conditioning stimulus after each treatment. Group differences are tested by t-test or χ^2-test. To quantify offset analgesia the decrease in eVAS from peak eVAS value to the eVAS nadir following the 1 ^{o}C decrease of the test stimulus is measured (ΔeVAS) corrected for the value of the peak eVAS ($\Delta eVAS_C$ = ΔeVAS/[peak eVAS]). $\Delta eVAS_C$ values observed after S(+)-ketamine and placebo treatment are compared using a linear mixed model. p-values < 0.05 were considered significant. Analysis is by SPSS for windows v 16.0.

7. Educational Grant

This study is supported by an educational grant from Grúnenthal GmbH, Aachen, germany.

8. References

Le Bars D, Dickenson AH, Besson JM. Diffuse noxious inhibitory controls (DNIC). I. Effects on dorsal horn convergent neurones in the rat. Pain. 1979; 6:283-304 (first description of DNIC)

Grill J and Coghill RC. Transient analgesia evoked by noxious stimulus offset. J Neurophysiol 2002; 87: 2205-2208.

Hatrick CT. Tapentadol immediate release for the relief of moderate-to-severe acute pain. Expert Opinion Pharmacother. 2009; 10: 2687-2696.

King et al. Deficiency in endogenous modulation of prolonged heat pain in patients with irritable bowel syndrome and temporomandibular disorder. Pain (2009) doi:10.1016/j.pain.2008.12.207

Pud et al. The methodology of experimentally induced diffuse noxious inhibitory control (DNIC)-like effect in humans. Pain (2009) doi:10.1016/j.pain.2009.02.015

Villanueva L. Diffuse noxious inhibitory control as a tool for exploring dysfunction of endogenous pain modelatory systems. Pain (2009) doi:10.1016/j.pain.2009.03.003

Wade E. et al. Tapentadol hydrochloride: A centrally acting oral analgesic. Clin Ther 2009; 31: 2804-2818.

Yarnitsky D. et al. Prediction of post-operative pain: pre-operative DNIC testing identifies patients at risk. Pain 2008; 138: 22-28.

Yelle MD et al. Offest analgesia: a temporal contrast mechanism for nociceptive information. Pain 2008; 134: 174-186.

***

9. Information for volunteers.

***** INFORMATIE VOOR PATIENTEN *****

Informatie voor vrijwilligers en patiÎnten die meedoen aan de TPT-studie; Locatie: Anesthesia & Pain Research Unit, LUMC, kamer K5-120

Versie 1, Sept 29, 2010

Beste meneer/mevrouw,

Nadat u reeds mondeling bent ingelicht over bovengenoemd onderzoek volgt hier een schriftelijke toelichting. Lees deze toelichting zorgvuldig, bespreek hem met vrienden en/of familie en neem bij vragen contact met ons op. Beslis dan of u wilt meedoen aan dit onderzoek. Deelname aan de studie leidt niet tot een klinisch voordeel voor u. Weigering om mee te doen aan de studie, of terugtrekking uit de studie (op welk moment dan ook) zal zonder enig effect zijn op uw huidige of toekomstige medische behandelingen.

Waarom dit onderzoek en hoe is het opgezet?
Chronische pijn is een sterk invaliderend proces dat behoudens de lichamelijke schade ook emotionele en zelfs economische consequenties heeft. In Nederland komt chronische pijn bij 1 op de 6 (16%) mensen voor en bij 1 op de 12 zelfs continu. Verschillende therapieÎn worden toegepast om chronische neuropathische pijn te behandelen. Geen van allen is afdoende effectief of heeft slechts een zeer kort effect. Een nieuw geneesmiddel genaamd tapentadol geeft veelbelovende resultaten in eerder uitgevoerd onderzoek naar chronische pijn. Het middel werkt op het centraal zenuwstelsel (net zoals morfine) en geeft hierdoor pijnstilling en mogelijk verbetering van functionaliteit. Mogelijk heeft tapentadol zelfs een bredere werking dan alleen een pijnstillend effect. In deze studie willen wij onze kennis met betrekking tot chronische pijn bij diabetes patiÎnten vergroten en bestuderen hoe het geneesmiddel Tapentadol de pijn beÔnvloedt.

Om dit te bereiken zullen we u een aantal warmtepijnprikkels toedienen. De prikkels duren ca. 30 seconde en u wordt gevraagd met behulp van een schuiflat de pijn te scoren gedurende de gehele 30 seconde die de pijnstimulus duurt. Alvorens we met het echte experiment starten zal het een en ander worden uitgelegd en geoefend. Hierna krijgt u via een infuus en via een tablet die oraal wordt ingenomen een medicijn toegediend. Aan het einde van de toediening en de periode hierop volgend worden opnieuw pijnprikkels aangeboden die u ook nu weer moet scoren. In totaal worden er drie verschillende medicijnen op verschillende dagen toegediend. De eerste is tapentadol, de andere zijn morfine en een placebo die dienen als controle.

Hoe wordt het onderzoek uitgevoerd
U wordt op drie afzonderlijke dagen getest. De gehele testperiode per dag is ca. 5 uur. Er zullen drie verschillende middelen worden getest, namelijk Tapentadol, morfine en een stof waarvan de werkzaamheid niet bekend is. Welk middel u wanneer krijgt toegediend wordt gebaseerd op toeval. We beginnen rond 8:30 en u kunt om ca. 13:30 weer naar

huis. De medicijnen worden als tablet via de mond toegediend.

Na een korte rustperiode gaan we oefenen met het scoren van de pijnprikkel. De temperatuur wordt overgebracht via een 3 bij 3 cm groot element dat op de huid van een arm wordt geplaatst. De temperatuur zal vanaf 32 oC snel oplopen tot 42 oC. Deze temperatuur wordt dan gehandhaafd gedurende ca. 6 seconde. U scoort de pijn door een schuifje te verplaatsen van 0 (= geen pijn) tot 10 (= meest denkbare pijn). Deze oefening zal een paar keer worden herhaald bij verschillende temperaturen.

De eerste pijntest is een hitteprikkel die eerst niet maar later wel wordt gecombineerd met het onderdompelen van een van uw voeten in een bad met koel water. Eerst zullen wij het onderdompelen oefenen. De watertemperatuur varieert tussen de 6 en 16 oC. De pijntest zal 3 maal worden getest zonder onderdompelen en driemaal met onderdompelen van de voet. De tweede pijntest is een variatie van de eerste maar wordt niet gecombineerd met het onderdompelen van de voet. De pijnprikkel is opnieuw ca. 30 seconde maar zal nu niet constant zijn. Opnieuw scoort u de pijn met behulp van de schuiflat. Ook deze prikkel wordt tweemaal herhaald. Verder zullen we u een aantal keer vragen hoeveel pijn u spontaan ondervindt (dus zonder pijnprikkel toediening).

Na een korte rustperiode slikt u een van de geneesmiddelen. Circa 1 uur na het doorslikken van het tablet zullen we opnieuw een aantal pijnprikkels toedienen om na te gaan hoe groot het effect van de pijnstilling is. Na het staken van de toediening van de pijnstillers herhalen we pijntesten zoals boven beschreven.

Wie doen er mee aan deze studie?
Aan deze studie doen gezonde vrijwilligers mee en patiÎnten die gediagnosticeerd zijn met diabetische neuropathie. Er worden geen andere patiÎntengroepen getest.

Belangrijk (1)
Alhoewel wij niet verwachten dat u thuis nog suf bent raden we u aan ës avonds niets te plannen en ook geen alcohol te drinken. Ook is het belangrijk om niet alleen naar huis te gaan maar zich door iemand anders te laten rijden of onder begeleiding met trein of bus naar huis te gaan.

Belangrijk (2)
Drink tijdens de week voor het onderzoek geen grapefruitsap. Gebruik 24 uur voorafgaande aan de studie geen alcohol, koffie, thee of chocolade. Eet of drink niets gedurende de 6 uur voorafgaande aan de studie.

Bijwerkingen
Na de toediening de geneesmiddelen kunnen zich bijwerkingen voordoen. Echter gezien de korte lage toe te dienen dosis verwachten wij dat deze mild zullen zijn. De beschreven bijwerkingen van morfine zijn: misselijkheid, braken, euforie, duizeligheid, verlaagde hartslag, verminderde ademhaling en een allergische reactie kan geven. Tapentadol kan duizeligheid, slaperigheid, misselijkheid, evenwichtsstoornissen en een droge mond veroorzaken. Mochten zich bijwerkingen voor doen, zullen deze indien mogelijk

behandeld worden.

De hitte pijnprikkel kan een niet pijnlijke rode verkleuring van de huid geven. Deze is van korte duur en verdwijnt na een aantal uren. De pijnprikkel kan geen verbranding veroorzaken.

Vrijwillige deelname
Uw medewerking aan dit onderzoek is vrijwillig. Als u toestemming geeft om aan dit onderzoek mee te doen, heeft u te allen tijde de vrijheid om op die beslissing terug te komen. U hoeft hier geen reden voor op te geven.

Betaling
De vergoeding bedraagt voor een volledig afgeronde sessie (1 dag) EURO 80.-. Besluit u eerder met de studie te stoppen dan wordt u naar rato betaald.

Vertrouwelijkheid van de gegevens
Alle gegevens worden onder code (d.w.z. zonder vermelding van uw naam en adres) verzameld en vertrouwelijk behandeld. De resultaten van dit onderzoek kunnen gebruikt worden in een wetenschappelijke publicatie, maar ook dan zijn de gegevens niet tot u als persoon herleidbaar.

Verzekering
Het ziekenhuis heeft een verzekering afgesloten waaruit eventuele schade als gevolg van het onderzoek betaald kan worden. Als u vindt dat u schade heeft ondervonden als gevolg van het onderzoek waaraan u meedoet (of heeft meegedaan) kunt u dit het beste met de onderzoeker bespreken.

Tot slot
U krijgt een kopie van het informatieformulier en het gesigneerde toestemmingsformulier mee naar huis. Mocht u naar aanleiding van deze informatie nog vragen hebben met betrekking tot dit onderzoek dan kunt u daarmee bij een van ons terecht.

Drs. M. Niesters, onderzoeker anesthesiologie
Prof dr Albert Dahan, anesthesioloog
Afdeling Anesthesiologie, LUMC, P5
Tel 071 526 2301 of 071 526 9111.

Ook kunt u contact op nemen met Dr. E. Sarton (LUMC, afdeling anesthesiologie, tel 071 526 2301). Zij is niet betrokken bij de uitvoering van deze studie maar wel op de hoogte van de gang van zaken. Het is mogelijk met haar de gehele procedure rustig door te spreken.

10. Informed Consent (Toestemmingsformulier) versie :1.0; datum: 1 mei - 2009

Toestemmingsformulier betreffende de DNIC studie

Ik ben naar tevredenheid over het onderzoek geïnformeerd. Ik heb de schriftelijke informatie goed gelezen. Ik ben in de gelegenheid geweest om vragen over het onderzoek te stellen. Mijn vragen zijn naar tevredenheid beantwoord.

Ik heb goed over deelname aan het onderzoek kunnen nadenken. Ik heb het recht mijn toestemming op ieder moment weer in te trekken zonder dat ik daarvoor een reden voor moet opgeven.

Ik ben geïnformeerd over de betaling en ik begrijp dat ik indien ik de studie niet voltooi naar rato zal worden betaald.

- **Ik geef toestemming voor deelname aan het onderzoek.**

Achternaam en voorletters : …………………………………………..

Geboortedatum : …………………………………………..

Handtekening : …………………………………………..

Datum: : …………………………………………..

- Ondergetekende verklaart dat de hierboven genoemde persoon zowel mondeling als schriftelijk over het bovenvermelde onderzoek is geïnformeerd. Hij/zij verklaart tevens dat een voortijdige beëindiging van de deelname door bovengenoemde persoon, van geen enkele invloed zal zijn op de zorg die hem of haar toekomt.

Naam : …………………………………………………

Functie : …………………………………………………

Handtekening : …………………………………………………

Datum: : …………………………………………………

11. Advertentietekst

Gezocht

Gezonde proefpersonen (18-75 jr) en patiënten met chronische pijn en diabetes die, voor een vergoeding, mee willen doen aan een onderzoek naar de effecten van een geneesmiddel op de pijnstilling.

Indien u belangstelling heeft, neem dan contact op met:
Dr. M. Niesters of prof. dr. A. Dahan

Afdeling Anesthesiologie van het LUMC
Tel: 071 526 2301

12. **Addendum**

Declaration by the Investigator

I have read this protocol and agree that it contains all the necessary details for carrying out this trial. I agree to personally conduct or supervise the trial as described and will complete the trial within the time designated. I verify that I am suitably qualified by my education, scientific medical training, and experience to conduct the trial. I also verify that I have had Good Clinical Practice training or, if training has not already been completed, agree to complete Good Clinical Practice training organized by the Sponsor. Documentation of my qualifications and professional affiliations are contained in my signed and dated current curriculum vitae.

I will provide the supplied copies of the protocol and all information relating to preclinical and prior clinical experience (e.g., Summary of Product Characteristics) to all staff in my unit who will participate in this trial. I will discuss this material with them to ensure that they are fully conversant with the medical treatment in and the conduct of the trial, and that they will handle the data and information generated in the trial confidentially. This distribution of copies of the protocol and all information relating to pre-clinical and prior clinical experience (e.g., Summary of Product Characteristics) and discussion of the material will be documented.

I agree not to start enrolling subjects until a duly appointed Independent Ethics Committee has issued a favorable opinion and the Competent Authorities have approved the trial.

I will conduct the trial according to the ethical principles that have their origin in the Declaration of Helsinki and that are consistent with Good Clinical Practice and applicable regulatory requirement(s) relating to clinical trials and the protection of subjects in the country in which the trial will be performed. All subjects will be comprehensively informed about the nature of the trial and of its investigational nature and that they may withdraw from the trial at any time. They will give their written consent to participate before entry into the trial. I will only use the information sheet and Informed Consent Form approved by the Sponsor and the Independent Ethics Committee that has reviewed this trial. I will supply the Sponsor with any material written by me (e.g., summary of trial, correspondence, etc) that has been given to the Independent Ethics Committee in support of the application.

I have read the Summary of Product Characteristics, including the potential risks and side effects of the Investigational Medicinal Products, and I agree to report adverse events that occur during the trial.

Where applicable, the information contained in the Case Report Forms will be transcribed from my records, reports, and manuscripts. The Case Report Form may be the original source document for certain items. Either I or an appointed person will attest to the authenticity of the data and accuracy and completeness of the transcriptions by signing the Case Report Form. I agree to the audit and monitoring procedures described in the protocol, which involve verification of trial records against the original records. I will make available additional background data from my records at the request of

government regulatory agencies, if allowed by the hospital or institution where the trial is conducted.

I understand that I am obliged to provide to the Sponsor for his or her unrestricted use the complete results and all data generated during the trial, and that all information concerning the Investigational Medicinal Products and Sponsor's activities, such as patents, formulae, manufacturing procedures, and basic, unpublished scientific data and information supplied by the Sponsor are confidential and are the exclusive property of the Sponsor.

I undertake only to use this information to conduct the trial and not to use it for any other purpose without the written agreement of the Sponsor.

I will supply the Sponsor with the trial data in such a way that the subjects cannot be personally identified.

Date: Leiden, September 29, 2010

Name: Prof. dr. A. Dahan

Signature:

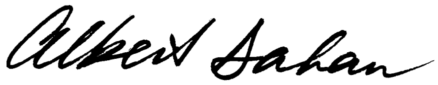

Supplement: S1 Protocol — (PDF) [file pone.0128997.s002.pdf]
